# Marital dissatisfaction and adverse health and psychosocial outcomes among community-dwelling persons 45 years and older in Thailand

**DOI:** 10.1080/21642850.2025.2589568

**Published:** 2025-11-23

**Authors:** Supa Pengpid, Karl Peltzer, André Hajek, Razak M. Gyasi

**Affiliations:** a Department of Health Education and Behavioral Sciences, Faculty of Public Health, Mahidol University, Bangkok, Thailand; b Department of Public Health, Sefako Makgatho Health Sciences University, Pretoria, South Africa; c Department of Healthcare Administration, College of Medical and Health Science, Asia University, Taichung, Taiwan; d Department of Psychology, University of the Free State, Bloemfontein, South Africa; e Department of Psychology, College of Medical and Health Science, Asia University, Taichung, Taiwan; f Department of Medical Research, China Medical University Hospital, China Medical University, Taichung, Taiwan; g Department of Health Economics and Health Services Research, University Medical Center Hamburg-Eppendorf, Hamburg Center for Health Economics, Hamburg, Germany; h African Population and Health Research Center, Nairobi, Kenya; i National Centre for Naturopathic Medicine, Faculty of Health, Southern Cross University, Lismore, NSW, Australia

**Keywords:** Adults 45 years and older, marital dissatisfaction, health outcomes, longitudinal study, Thailand

## Abstract

**Background:**

Only a small number of research have evaluated marital dissatisfaction and adverse health and psychosocial outcomes in lower-resourced countries. The aim of the study was to estimate the long-term associations of marital dissatisfaction with adverse health outcomes using four waves (7 years) of the national community-dwelling Health, Aging, and Retirement in Thailand (HART) study in people 45 years and older (also stratified by sex) from 2015 to 2022.

**Methods:**

HART data from the 2015, 2017, 2020, and 2022 waves were analysed, including community-dwelling individuals aged 45 and above (average age 65.1 years) who indicated marital dissatisfaction (analytic pooled sample: *n* = 10790 observations). Marital dissatisfaction was measured using validated scales. The time-variant causes and outcomes were evaluated using linear fixed effects regression. To determine the hazard ratios (HRs) and evaluate the associations between marital discontent and 7-year mortality in the entire sample, a Cox proportional hazards regression model was applied.

**Results:**

The average marital dissatisfaction score (0−10) was 1.8 (SD = 2.3). Fixed effects regressions showed that marital dissatisfaction was associated with low self-reported mental health, depressive symptoms, low quality of life, loneliness, functional limitations, poor self-rated physical health, low mastication, low exercise frequency, low meal skipping, and high subjective life expectancy. In addition, marital dissatisfaction was associated with all-cause mortality.

**Conclusions:**

Our knowledge of the connection between marital discontent and negative health outcomes—four markers of physical and mental health, including mortality—is improved by this longitudinal study. Measures of marriage happiness and the health advantages of marital education programs for couples should be part of health promotion initiatives for the aging population as a whole.

## Introduction

According to developmental research, age-related transitional times (such as becoming parents or retiring) require modifications and accommodations within the marriage dyad, so marital happiness varies over the life cycle (Gagnon et al., 1999). High stress levels brought on by various role responsibilities and family changes may be negatively associated with marital satisfaction as couples get closer to midlife (Gagnon et al., 1999). Marital stress may be associated with the normal deterioration in self-rated health over time, according to growth curve data from a nationwide longitudinal survey (Umberson et al., 2006). Given that marital stress appears to have a cumulative impact on health over time, making people more susceptible to marital strain as they age, these findings are consistent with earlier theoretical work on cumulative adversity (Umberson et al., 2006). Recent studies highlight that the health implications of marital satisfaction may differ by gender, with some studies suggesting that older women and men may respond differently to marital stress, dissatisfaction, and emotional support within marriage (Kim & Kwon, 2023).

The population of Thailand is rapidly aging, and life expectancy at 60 rose from 20.2% to 21.9% between 2000 and 2015 (Anantanasuwong, 2021). In 2022, 13 million people aged ≥ 60 constitute about 19% of the Thai population, which is expected to increase further to 19 million, accounting for 31.4% in 2042 (Department of Older Persons, 2023). In a case-control study of major depressive disorder patients (60 years and older) at a psychiatric outpatient department in Bangkok, compared to the control group, the depressed group's marital satisfaction levels were lower (Suksathien & Lotrakul, 2009), and in a study among health care staff in Samutprakarn Province, Thailand, 55.2% reported to have high marital quality (Sorajtanon et al., 2011). This study was prompted by the lack of longitudinal data regarding the association between poor health outcomes and marital dissatisfaction among middle-aged and older adults in low- and middle-income countries (LMICs), such as Thailand.

Higher marital quality was linked to psychological well-being in both cross-sectional and longitudinal studies that examined the relationship between psychological well-being indicators and marital quality (e.g. self-esteem, life satisfaction, happiness, and symptoms of anxiety and/or depression) (Korporaal et al., 2013; Proulx et al., 2007). Research on longitudinal studies is less clear, despite the seeming strong evidence for a cross-sectional association between depression and marital quality. This is especially true for older persons, about whom there are few research calling for additional studies and the clinical applications of this field's research (Goldfarb & Trudel, 2019). For example, in a longitudinal study in U.S.A., there was a continuous correlation between rates of marital discontent and later reports of depression symptoms for men (Woods et al., 2019). According to Bookwala (2005), there was a distinct correlation between physical health and higher levels of negative marital behaviors, which indicated worse subjective health, physical disabilities, chronic health problems, and more physical symptoms. Greater marital quality was related to better health (better self-rated health and/or lower self-rated symptoms) (Korporaal et al., 2013; Robles et al., 2014), including lower risk of mortality (Lev-Ari et al., 2021; Robles et al., 2014; Whisman et al., 2018). In a longitudinal study in South Korea, marital discontent was positively associated with functional disability and a negatively associated with grip strength, mastication, eyesight, and hearing (Kim & Kwon, 2024). In a longitudinal study in the U.S.A., analyzes revealed that improvements in both spouses' good marital quality were independently linked to improvements in their self-rated health in midlife and old age and decreases in disability in old age. For ageing adults, decreases in functional limits were similarly associated with increases in positive marital quality (Choi et al., 2016).

In terms of health risk behavior, marital dissatisfaction has been found to be associated with lack of exercise, excessive drinking and smoking (Azizi et al., 2024), and poor dietary behavior (Omidvar et al., 2018). The information that is now available indicates that problematic drinking and marital distress frequently worsen one another, creating a harmful cycle, even if the causal relationships between the two issues are complicated and only poorly understood (Rodriguez et al., 2014).

Considering the paucity of information on this topic, particularly with regard to longitudinal research conducted in LMICs, our study sought to investigate the associations of marital dissatisfaction with adverse health and psychosocial outcomes longitudinally using four waves (7 years) of the national community-dwelling Health, Aging, and Retirement in Thailand (HART) study in people 45 years and older (also stratified by sex) from 2015 to 2022. Understanding how marital dissatisfaction contributes to adverse health and psychosocial outcomes is crucial for creating therapies meant to help older persons.

## Methods

### Sample

Four waves of health, aging, and retirement in Thailand (HART) studies conducted in 2015, 2017, 2020, and 2022 were analyzed. One person (≥45 years) was randomly selected from each household (as the inclusion criterion) and questioned at home using a multi-stage national sample method; more information is provided elsewhere (Anantanasuwong et al., 2019). Only married participants who reported marital satisfaction levels were included in the sample for this research. 10790 observations from four study evaluations in 2015, 2017, 2020, and 2022 who expressed marital dissatisfaction made up the analytical pooled sample; 229 of them had passed away, and 38 had declined. The individuals lost to follow-up in comparison to those who remained in the study did not significantly differ on living alone, residence status, quality of life, exercise frequency, alcohol use frequency, marital dissatisfaction, subjective economic status, religious involvement, and number of chronic conditions. Loss to follow-up was higher in women than men, those who were working, had higher education, had higher loneliness, higher meal skipping frequency, higher subjective life expectancy, lower depressive symptoms, lower self-rated physical health, lower number of physical limitations, lower mastication, and younger age.

### Ethics statement

The study received ethical approval from the ‘Ethics Committee in Human Research, National Institute of Development Administration—ECNIDA (ECNIDA 2020/00012)’, and participants provided written informed con-sent. All experiments were performed in accordance with relevant guidelines and regulations (such as the Declaration of Helsinki).

## Measures

Matching our fixed effects regression analyzes, all considered variables were time-varying.

## Dependent variables

Depression symptoms were measured using the Center for Epidemiologic Studies Depression Scale (CES-D−10), which has a total score range of 0 to 30. Higher scores on the scale correspond to more depressive symptoms (Andresen et al., 1994). 0.69 was Cronbach's alpha.

On a visual analog scale of ‘0 (extremely poor) to 10 (excellent)’, the self-rated mental health status was measured with the item, ‘In general, how would you rate your mental health status?’ Research has demonstrated the validity of single item self-rated health metrics (Schnittker & Bacak, 2014).

Quality of life (QoL) was assessed with the item, ‘Overall, how satisfied are you with your quality of life?’ reported on a ‘0 ( = very poor) to 10 ( = excellent) scale’. The validity of single-item QoL tests has been established (Zimmerman et al., 2006).

*Loneliness* was measured using the CES-D−10 item (Andresen et al., 1994), ‘In the past week, how often did you experience feeling lonely?’ defined as ‘almost always (5−7 days), often (3−4 days) or sometimes (1−2 days)’ = 1 and ‘very rarely (less than one day) or none’ = 0. Items were reverse scored so that higher scores reflected higher loneliness. There are strong relationships between this loneliness score and other loneliness measures that use numerous items (Mund et al., 2022), and ‘performs similarly to other loneliness measures’ (Newmyer et al., 2021).

The question, ‘In general, how would you rate your physical health status?’ was used to construct the self-rated physical health status, given a score between 0 (extremely poor) and 10 (outstanding) (Pengpid & Peltzer, 2023).

*Functional limitations* were assessed with a modified 5-item Activities of Daily Living (ADL) scale (dress, wash, eat, bathing or showering, and other) (Katz et al., 1964). Response options were 0) ‘Can do every step myself without needing help,’ 1) ‘Need help sometimes with some steps,’ 2) ‘Need help with some steps,’ 3) ‘Need help sometimes with every step,’ and 4) ‘Need help every time with every step.’ Higher scores indicated higher ADL; total scores varied from 0 to 20. It has a Cronbach's *α* of 0.95.

*Mastication* was assessed with two questions, ‘What is your ability to chew sticky/hard/crispy things such as meat, guava, apple?’ a) wearing dentures and b) not wearing dentures. Response options were ‘1) Very bad, 2) A bit bad, 3) Average, 4) Fairly good and 5) Very good.’ Scores (2−10) of the two questions were summed, with higher scores indicating better mastication (Kim & Kwon, 2024).

To determine the frequency of *physical activity* or exercise, participants were asked*,* ‘How often do you exercise?’ (‘1 = 0 days, 2 = 1−2 days, 3 = 3−4 days, 4 = 5−6 days or 5 = 7 days per week’).

Alcohol use frequency 0 = none, 1 = 'Drink very little (e.g. drink no more than 1 small can of beer or no more than 1 glass of wine/spirits)', 2 = 'Drink lightly (e.g. drink 1 - 2 cans of beer or 1 - 2 glasses of wine/spirits) ’, 3 = ‘Drinks quite a bit (e.g. drinks about 3 - 4 cans of beer or 3 - 4 glasses of wine/spirits)’, 4 = ‘Drinks quite a lot (e.g. drinks 5 - 6 cans of beer or 5 - 6 glasses of wine/spirits) glasses)’, 5 = ‘Drinks a lot (such as drinking more than 6 small cans of beer or more than 6 glasses of wine/spirits)’. The higher the score (0−5), the higher the frequency of alcohol use.

Tobacco use was evaluated using the inquiry, ‘Have you ever smoked cigarettes?’ (response options: ‘1 = yes, and still smoke now, 2 = yes, but quit smoking, and 3 = never’), and those who were currently smoking were asked about the average amount smoked per day (number).

*Meal skipping* (0−6) was assessed with the question: ‘How many meals have you had in the last 2 days?’ ‘Yesterday (breakfast, lunch, dinner; yes/no) and the day before yesterday (breakfast, lunch, dinner; yes/no).’ (Pengpid et al., 2024).

Subjective *life expectancy* was measured using the question:

“*What are the chances (from 0–100%) that you will live to be x years or more? For individuals aged 65 years or less, x = 75 years or more; for those aged 66–69 years, x = 80 years or more; for those aged 70–74 years, x = 85 years or more; for those aged 75–79 years, x = 90 years or more; for those aged 80–84 years, x = 95 years or more; for those aged 85−94 years in wave 1 and 85−89 years in wave 2, x = 100 years or more, for those aged 95–99 years in wave 1 and wave 2 90−99 years, x = 105 years or more, and for those aged 100−105 years, x 110 years or more*” (Kim & Kim, 2017).

Survival and exposure to death are used to calculate mortality. To determine the interviewee's survival status, the question was whether they died in the 2015 wave or lived in the waves of 2017, 2020, and 2022.

## Independent variables

The question was used to measure marital satisfaction, ‘In general, how satisfied are you with your spouse?’ on a scale of 0 to 10, where 10 represents the highest level of satisfaction and 0 the lowest. Higher scores denoted more marital discontent since the items were reverse graded (Kim & Kwon, 2024).

## Control variables

Age, work status (yes/no), living situation (living alone/living with others), and subjective economic status were among the sociodemographic characteristics that varied over time. ‘How satisfied are you with your economic situation?’ was the question used to assess the latter. Higher economic status is indicated by higher scores, which range from 0 to 10. Time-constant factors including sex, urban/rural residency, and educational achievement were utilized as sample descriptions, and sex was used in a gender-stratified model.

Healthcare providers identified the following chronic conditions (0–15): diseases of the liver, gastrointestinal tract, kidneys, lungs, prostate, uterine or ovarian, cancer, diabetes, hypertension, bone diseases, cardiovascular disease, mental or emotional disorders, brain disease or dementia, visual or auditory impairment, and self-reported urine incontinence during the previous month.

Assessment of *religious involvement* included 1) ‘Making merit and giving alms according to respected religious principles’, 2) ‘Prayer in the morning/before going to bed,’ 3) ‘Performing merit-making activities at religious places according to the religions that the interviewees respect on important religious days,’ and 4) ‘Observing important religious days that the interviewee respects.’ The Cronbach alpha was 0.83, and the response choices included a total of 0–12, with 0 denoting never and 3 denoting always. Higher scores indicated stronger religious commitment.

## Statistical analysis

The sample was described using descriptive statistics. The percentage differences by gender were calculated using chi-square and t-tests. The longitudinal relationships between time-varying independent factors and time-varying dependent variables were estimated using linear fixed effects (FE) regressions for the four study waves conducted between 2015 and 2022. The Hausman specification test between the fixed-effects and random-effects models validated the selection of FE regression (for the 12 dependent variables: (35.06−417.38) *p* < 0.001). Because all person-level variability is explained by individual fixed effects, this model is particularly effective at reducing biases brought on by systematic relationships between explanatory variables and time-constant factors (both observed and unobserved), such as genetics (Brüderl & Ludwig, 2015). Therefore, the use of FE regressions significantly reduces the issue of unobserved heterogeneity. Based on previous research, time-varying determinants were incorporated (Hwang et al., 2024; Kim & Kwon, 2024; Kim, 2023). Sex and education are examples of time-invariant variables that cannot be incorporated into linear FE regressions as major effects. Consequently, education was utilized for descriptive purposes and sex, a time-constant component, was used to stratify the regression models. *P* < 0.05 was deemed significant.

Missing values for all variables were between 0 and 2.0%. It is advised against using imputation in this situation because low levels of missing information typically do not substantially affect the results (Allison, 2001; Van Buuren, 2012). Further, FE lessens the risk of bias from missing values by reducing the risk from unobserved time-constant variables (Brüderl & Ludwig, 2015; Brüderl, 2010; Wooldridge, 2010). Listwise deletion is used in fixed effects regression to deal with missing values. We computed cluster-robust standard errors. To determine the hazard ratios (HRs) and evaluate the relationships between marital discontent and 7-year mortality in the entire sample, a regression model using Cox proportional hazards was employed. For statistical analysis, StataSE 18.0 (College Station, TX, U.S.A.) was utilized.

## Results

### Features of the sample

There were 10790 observations in the combined analytical sample. The average age of the pooled sample was 65.1 years (range: 80−107 years), 23.5% had completed more than basic school, 51.6% lived in an urban location, and 7.5% lived alone. The average marital dissatisfaction score (0−10) was 1.8 (SD = 2.3), which was significantly higher in women than in men. Educational level differed by sex, and residence status did not differ by sex. Control variables, including age, work status, number of chronic conditions, religious involvement, and subjective economic status differed by sex. Regarding dependent variables, depressive symptoms, loneliness, alcohol use frequency, number of cigarettes per day, meal skipping, and mortality differed by sex (see Table 1).

**Table 1.** Analytic sample characteristics, pooled waves (1, 2, 3 and 4), HART 2015−2022.

Variable (range)	Total	Male	Female	*p*-value^a^
Observations	10790	5660	5130	
	*N* (%)/Mean (SD)	*N* (%)/Mean (SD)	*N* (%)/Mean (SD)	
**Dependent variable**				
Depressive symptoms (0−30)	4.57 (3.6)	4.46 (3.5)	4.70 (3.7)	<0.001
Self-rated mental health status (0−10)	8.23 (1.6)	8.25 (1.6)	8.20 (1.7)	0.155
Quality of life (0−10)	8.05 (1.5)	8.05 (1.5)	5.04 (1.5)	0.777
Loneliness (1−4)	2.76 (1.1)	2.72 (1.1)	2.81 (1.1)	<0.001
Self-rated physical health (0−10)	7.41 (1.7)	7.42 (1.7)	7.40 (1.8)	0.491
Functional limitations (0−20)	0.20 (1.4)	0.22 (1.4)	0.18 (1.3)	0.137
Mastication (0−5)	3.33 (1.2)	3.35 (1.2)	3.32 (1.2)	0.071
Exercise frequency (1−5)	2.55 (1.7)	2.58 (1.7)	2.52 (1.7)	0.057
Alcohol use frequency (0−5)	0.45 (1.2)	0.72 (1.6)	0.16 (0.6)	<0.001
Number of cigarettes per day	1.53 (4.7)	2.79 (6.1)	0.13 (1.2)	<0.001
Meal skipping frequency	0.33 (0.9)	0.30 (0.8)	0.36 (0.9)	<0.001
Subjective life expectancy (0−100)	66.8 (27.9)	66.4 (28.2)	67.2 (27.8)	0.132
Mortality	229 (2.1)	48 (0.8)	181 (3.5)	<0.001
**Independent variable**				
Marital dissatisfaction (0−10)	1.8 (2.3)	1.5 (1.9)	2.1 (2.6)	<0.001
**Control variable**				
Age (45−107 years)	65.1 (11.0)	66.5 (11.1)	63.5 (10.6)	<0.001
Subjective economic status (0−10)	6.64 (1.9)	6.67 (1.9)	6.62 (1.9)	0.161
Work status (working)	49.5	53.6	45.1	<0.001
Living arrangement (living alone)	7.5	7.2	7.8	<0.001
Number of chronic conditions (0−15)	1.17 (1.3)	1.13 (1.3)	1.22 (1.3)	<0.001
Religious involvement (0−12)	6.20 (3.4)	5.99 (3.5)	6.43 (3.2)	<0.001
**Time-invariant variables**				
Education (>primary)	23.5	26.9	19.8	<0.001
Residence (urban)	51.6	51.8	51.3	0.691

^a^ based on Chi-square tests or t-tests.

### Marital dissatisfaction and mental health

Marital discontent was significantly correlated with depression symptoms in men (*β* 0.35, *p* < 0.001), women (*β* 0.16, *p* < 0.001), and the entire sample (*β* 0.25, *p* < 0.001), according to the results of the linear FE regression. Additionally, marital dissatisfaction was associated with poor self-rated mental health among women (*β* −0.17, *p* < 0.001), men (*β* −0.35, *p* < 0.001), and the entire sample (*β* −0.25, *p* < 0.001). Moreover, marital dissatisfaction was associated with poor quality of life among women (*β* −0.16, *p* < 0.001), men (*β* −0.28, *p* < 0.001), and the entire group (*β* −0.22, *p* < 0.001). Lastly, marital dissatisfaction was associated with loneliness in men (*β* 0.02, *p* < 0.001) and the overall sample (*β* 0.02, *p* < 0.001), but not women. In the overall sample, adding marital dissatisfaction to the model explains an additional 3%, 8%, 7% and 0.1% of the within-person variance in depressive symptoms, self-rated mental health, quality of life and loneliness, respectively (see Table 2).

**Table 2.** Associations with mental health from fixed-effects regression analyzes (waves 1, 2, 3, and wave 4).

	Depressive symptoms		Self-rated mental health status		Quality of life		Loneliness	
Variable	BC (CRSE)^a^	*p*-value	BC (CRSE)^a^	*p*-value	BC (CRSE)^a^	*p*-value	BC (CRSE)^a^	*p*-value
Marital dissatisfaction-all	0.25 (0.03)	<0.001	−0.25 (0.01)	<0.001	−0.22 (0.01)	<0.001	0.02 (0.004)	<0.001
Constant	19.56 (0.96)	<0.001	8.15 (0.40)	<0.001			1.07 (0.14)	<0.001
Observations	10554		10570		10570		10570	
Individuals	5768		5776		5776		5776	
R^2^	0.08		0.12		0.18		0.01	
R^2b^	0.05		0.04		0.11		0.009	
Marital dissatisfaction-men	0.35 (0.04)	<0.001	−0.32 (0.02)	<0.001	−0.28 (0.02)	<0.001	0.02 (0.006)	<0.001
Constant	18.32 (1.22)	<0.001	8.64 (0.55)	<0.001	8.29 (0.48)	<0.001	0.93 (0.19)	<0.001
Observations	5505		5511		5511		5511	
Individuals	2888		2891		2891		2891	
R^2^	0.09		0.16		0.21		0.02	
R^2b^	0.05		0.04		0.11		0.007	
Marital dissatisfaction-women	0.16 (0.04)	<0.001	−0.17 (0.02)	<0.001	−0.16 (0.01)	<0.001	0.01 (0.006)	0.064
Constant	22.65 (1.38)	<0.001	7.69 (0.62)	<0.001	6.09 (0.54)	<0.001	1.26 (0.22)	<0.001
Observations	5049		5059		5059		5059	
Individuals	2930		2935		2935		2935	
R^2^	0.09		0.10		0.16		0.01	
R^2b^	0.06		0.05		0.12		0.009	

BC = Beta Coefficient; CRSE = Cluster Robust Standard Errors;

^a^ adjusted for age, subjective economic status, work status, living arrangement, religious involvement, number of chronic diseases, and study wave;

^b^ R^2^ for the model for control variables.

### Marital dissatisfaction and physical health

Linear FE regression showed that there was a significant relationship between marital dissatisfaction and poor physical health status in men (*β* −0.18, *p* < 0.001), women (*β* −0.09, *p* < 0.001), and the overall group (*β* −0.13, *p* < 0.001). In addition, there was a significant correlation between marital dissatisfaction and functional limitations in the entire group (*β* 0.05, *p* < 0.001), among men (*β* 0.06, *p* < 0.001), and among women (*β* 0.05, *p* < 0.001). Furthermore, marital dissatisfaction was associated with poor mastication in the whole group (*β* −0.02, *p* = 0.017) and among women (*β* −0.02, *p* = 0.035). In the overall sample, adding marital dissatisfaction to the model explains an additional 3%, 1%, and 1% of the within-person variance in self-rated physical health status, functional limitations, and mastication, respectively (see Table 3).

**Table 3.** Associations with physical health from fixed-effects regression analyzes (waves 1, 2, 3 and wave 4).

	Self-rated physical health status		Functional limitations		Mastication	
Variable	BC (CRSE)^a^	*p*-value	BC (CRSE)^a^	*p*-value	BC (CRSE)^a^	*p*-value
Marital dissatisfaction-all	−0.13 (0.01)	<0.001	0.05 (0.01)	<0.001	−0.02 (0.009)	0.017
Constant	5.95 (0.40)	<0.001	−2.00 (0.34)	<0.001	5.52 (0.28)	<0.001
Observations	10570		10570		10570	
Individuals	5776		5776		5778	
R^2^	0.11		0.03		0.07	
R^2b^	0.08		0.02		0.06	
Marital dissatisfaction-men	−0.18 (0.02)	<0.001	0.06 (0.02)	<0.001	−0.01 (0.01)	0.387
Constant	6.10 (0.56)	<0.001	−1.52 (0.51)	0.003	5.49 (0.38)	<0.001
Observations	5511		5511		5511	
Individuals	2891		2891		2891	
R^2^	0.12		0.03		0.09	
R^2b^	0.08		0.02		0.08	
Marital dissatisfaction-women	−0.09 (0.02)	<0.001	0.05 (0.01)	<0.001	−0.02 (0.01)	0.035
Constant	5.65 (0.62)	<0.001	−2.53 (0.48)	<0.001	6.14 (0.41)	<0.001
Observations	5039		5059		5059	
Individuals	2935		2935		2935	
R^2^	0.10		0.03		0.06	
R^2b^	0.08		0.02		0.04	

BC = Beta Coefficient; CRSE = Cluster Robust Standard Errors;

^a^ adjusted for age, subjective economic status, work status, living arrangement, religious involvement, number of chronic diseases, and study wave;

^b^ R^2^ for the model for control variables.

### Marital dissatisfaction and health risk behavior

The results of linear FE regression indicated a significant relationship between marital dissatisfaction and low exercise frequency in the entire group (*β* −0.07, *p* < 0.001), as well as in men (*β* −0.10, *p* < 0.001) and women (*β* −0.05, *p* < 0.001). Additionally, marital dissatisfaction was associated with low meal skipping among men (*β* −0.03, *p* < 0.001) and the overall group (*β* −0.02, *p* = 0.004), but not among women. In addition, marital dissatisfaction was not significantly associated with alcohol use frequency and number of cigarettes smoked in the entire sample and both men and women. In the overall sample, adding marital dissatisfaction to the model explains an additional 0.6%, and 1% of the within-person variance in exercise frequency and meal skipping frequency, respectively (see Table 4).

**Table 4.** Associations with health risk behavior from fixed-effects regression analyzes (waves 1, 2, 3, and wave 4).

	Exercise frequency		Alcohol use frequency		Number of cigarettes on average per day		Meal skipping frequency	
Variable	BC (CRSE)^a^	*p*-value	BC (CRSE)^a^	*p*-value	BC (RCSE)^a^	*p*-value	BC (CRSE)^a^	*p*-value
Marital dissatisfaction-all	−0.07 (0.01)	<0.001	0.003 (0.008)	0.680	−0.04 (0.03)	0.258	−0.02 (0.006)	0.004
Constant	2.62 (0.03)	<0.001	1.95 (0.27)	<0.001	−0.80	0.474	−1.42 (0.21)	<0.001
Observations	10570		10482		10570		10362	
Individuals	5776		5743		5776		5679	
R^2^	0.009		0.009		0.003		0.03	
R^2b^	0.003		0.008		0.002		0.02	
Marital dissatisfaction-men	−0.10 (0.02)	<0.001	0.009 (0.01)	0.523	−0.06 (0.07)	0.391	−0.03 (0.01)	<0.001
Constant	3.21 (0.62)	<0.001	3.29 (0.46)	<0.001	−1.17 (0.05)	0.562	−1.68 (0.30)	<0.001
Observations	5511		5439		5511		5441	
Individuals	2891		2864		2891		2849	
R^2^	0.02		0.02		0.005		0.03	
R^2b^	0.005		0.001		0.004		0.02	
Marital dissatisfaction-women	−0.05 (0.02)	0.006	−0.0009 (0.006)	0.895	−7.1 (0.01)	0.999	−1.32 (0.31)	0.535
Constant	1.81 (0.66)	0.006	0.43 (0.23)	0.064	0.02	0.966	−1.32 (0.31)	<0.001
Observations	5059		5043		5059		4952	
Individuals	2935		2929		2935		2879	
R^2^	0.01		0.003		0.02		0.03	
R^2b^	0.008		0.003		0.004		0.02	

BC = Beta Coefficient; CRSE = Cluster Robust Standard Errors;

^a^ adjusted for age, subjective economic status, work status, living arrangement, religious involvement, number of chronic diseases, and study wave;

^b^ R^2^ for the model for control variables.

### Marital dissatisfaction and subjective life expectancy and mortality

FE regression results showed a significant correlation between increasing marital dissatisfaction and decreasing subjective life expectancy in men (*β* −0.12, *p* < 0.001), women (*β* −0.11, *p* < 0.001), and the entire sample (*β* −0.11, *p* < 0.001). In the overall sample, adding marital dissatisfaction to the model explains an additional 3% of the within-person variance in subjective life expectancy.

Additionally, among women (HR: 1.08, 95% CI: 1.02−1.14) and the whole group (HR: 1.09, 95% CI: 1.03−1.14), but not among men, increases in marital dissatisfaction were substantially linked to mortality (see Table 5).

**Table 5.** Associations with subjective life expectancy from fixed-effects regression analyzes (waves 1, 2, 3, and wave 4) and hazard ratios of all-cause mortality.

	Subjective life expectancy		All-cause mortality	
Variable	BC (CRSE)^a^	*p*-value	HR (95% CI)^a^	*p*-value
Marital dissatisfaction-all	−0.11 (0.02)	<0.001	1.09 (1.03 to 1.14)	0.002
Constant	−5.61 (0.72)	<0.001		
Observations	10570			
Individuals	5776			
R^2^	0.08			
R^2b^	0.05			
Marital dissatisfaction-men	−0.12 (0.03)	<0.001	1.12 (0.99 to 1.27)	0.070
Constant	−5.62 (0.99)	<0.001		
Observations	5511			
Individuals	2891			
R^2^	0.08			
R^2b^	0.05			
Marital dissatisfaction-women	−0.11 (0.03)	<0.001	1.08 (1.02 to 1.14)	0.010
Constant	−6.52 (1.10)	<0.001		
Observations	5059			
Individuals	2935			
R^2^	0.08			
R^2^^b^	0.05			

BC=Beta Coefficient; CRSE= Cluster Robust Standard Errors; HR=Hazard Ratio;

^a^ adjusted for age, subjective economic status, work status, living arrangement, religious involvement, number of chronic diseases, and study wave.

^b^ R^2^ for the model for control variables.

Adding interaction variables to the primary models for sex, education, and residency status was the last stage. The interaction terms, however, fell short of statistical significance.

## Discussion

This study's objective was to make the initial estimation of the associations of marital dissatisfaction with adverse health and psychosocial outcomes longitudinally using four waves (7 years) of the national community-dwelling HART study in Thailand. FE regressions showed that marital dissatisfaction was associated with low self-reported mental health, depressive symptoms, low quality of life, loneliness, functional limitations, poor self-rated physical health, low mastication, low exercise frequency, low meal skipping, and high subjective life expectancy. In addition, marital dissatisfaction was associated with all-cause mortality. In the overall sample, adding marital dissatisfaction to the model explains an additional 3%, 8%, and 7% of the within-person variance in psychosocial health (depressive symptoms, self-rated mental health, quality of life, respectively), and adding marital dissatisfaction to the model explains an additional 3% of the within-person variance in self-rated physical health status and in subjective life expectancy. The marital dissatisfaction score (1.8) in this study was lower than in ageing South Korea (3.0) using a similar assessment measure (Kim & Kwon, 2024). In a study comparing Thai couples with Taiwanese couples (Taiwan being a high-income country like South Korea) when compared to Taiwanese couples, Thai couples expressed greater marital satisfaction (Huang et al., 2020).

Consistent with various studies (Downward et al., 2022; Goldfarb & Trudel, 2019; Korporaal et al., 2013; Proulx et al., 2007; Woods et al., 2019), this study showed an association between marital dissatisfaction and poor mental health (loneliness, low self-rated mental health, depression symptoms, and a low quality of life). According to the marital crisis model (Williams et al., 2010), marital discontent brought on by stress, disagreements, and other unpleasant feelings may worsen mental health. According to socioemotional selectivity theory, older people who restrict their social networks and concentrate more on their marriage may benefit from positive marital quality (Carstensen, 2021; Nazario-Acevedo et al., 2024).

In terms of adverse physical health outcomes, this study found, in agreement with previous studies (Bookwala, 2005; Choi et al., 2016; Kim & Kwon, 2024; Korporaal et al., 2013; Robles et al., 2014), that marital dissatisfaction was associated with poor self-rated physical health, functional disability and low mastication. Furthermore, consistent with various studies (Lev-Ari et al., 2021; Robles et al., 2014; Whisman et al., 2018), this study found that marital dissatisfaction increased the risk of mortality. Similarly, we found that marital dissatisfaction was associated with low subjective life expectancy. The model of stress processes (Pearlin et al., 1981) states that marital dissatisfaction is a life-course stressor that can be associated with physical health through physiological and psychological pathways. It can accelerate immunological dysregulation and result in functional limitations, for example (Kim & Kwon, 2024). Findings suggest that reducing marital dissatisfaction may increase longevity.

In terms of health risk behavior, we found in line with former studies (Azizi et al., 2024) that marital dissatisfaction was associated with physical inactivity. However, unlike some previous research (Azizi et al., 2024; Rodriguez et al., 2014), higher levels of alcohol and smoking were not significantly correlated with increases in marital dissatisfaction. Previous research among pregnant women found that marital dissatisfaction was associated with poor dietary behavior (Omidvar et al., 2018), while in our study, no significant association was found for meal skipping among women but an inverse association with meal skipping among men. Health behaviors like sleep, food, and exercise, as well as cognitive and affective processes, can be associated by marital stress (Slatcher & Schoebi, 2017). Increasing health care workers' knowledge and comprehension of the relational context in which health issues frequently arise could ultimately lead to a rise in the creation and adoption of couples-based therapies (Robles et al., 2014).

### Study strengths and limitations

The study used known measures from the Korean Longitudinal Study of Aging (KLoSA) and the Health and Retirement Study (HRS), and it included a 4-wave longitudinal, national community-dwelling population. Using panel FE regression models, we reduced the problem of unobserved heterogeneity. But depending on individual differences in FE models may reduce statistical power, which may have been the case for some of our outcomes, e.g. on substance use. Although the use of fixed effects models helps control for time-invariant individual characteristics, it does not account for time-varying unmeasured variables that may simultaneously affect both marital dissatisfaction and health outcomes. For instance, changes in financial status, caregiving responsibilities, major life events, or unmeasured aspects of the marital relationship (e.g. partner's health or external stressors) could confound the associations observed in the study (Kim & Kwon, 2023). The fact that all data were evaluated using self-report is one study drawback; objective measurements should be included in future evaluations. Marital dissatisfaction was only measured with a single item, and future studies may include multidimensional scales of marital quality. In addition, several concepts were measured with a single item, such as loneliness, quality of life, self-rated mental health, and self-rated physical health status, and future studies may include multi-item measures. Yet, these single-item measures have been found valid and comparable with multi-item measures (Cheung & Lucas, 2014; Lucas & Donnellan, 2012; Mund et al., 2022), Newmyer et al., 2021). Although efforts have been made to control for a number of time-varying confounders, reverse causality cannot be ruled out. Some of the variables that could be associated with marital dissatisfaction, such as cognitive function (Kim & Hwang, 2024) and grip strength (Kim & Kwon, 2024), were not assessed and should be included in future studies. A further limitation is that the data relies on a single source in data collection (e.g. self-reports of a single person) rather than several people (e.g. dyads) or objective data.

## Conclusions

By taking into account a variety of detrimental health consequences, the study contributes to the body of information on the health and quality of marriage in older persons. Marital dissatisfaction was associated with four poor mental health indicators and four poor physical health incidators, including mortality. Strategies to reduce marital dissatisfaction, such as measures that improve partners' ability to communicate and agree and social assistance programmes for functionally limited groups, may help reduce adverse health outcomes.

Participants provided signed informed consent, and the study's protocol was approved by the National Institute for Development Administration's Human Research Ethics Committee (ECNIDA 2020/00012).

## Data Availability

Data is publicly available at Health, Aging, and Retirement in Thailand (HART): https://hart.nida.ac.th/download-center/.
